# Impacts of sarcopenia on adverse events and prognosis in Chinese patients with esophageal cancer undergoing chemoradiotherapy

**DOI:** 10.3389/fnut.2025.1523674

**Published:** 2025-02-20

**Authors:** Jiaoyue Qu, Yang Liu, Yin Yuan, Zhao Yu, Jianming Ding, Zelai He, Gengming Wang

**Affiliations:** Department of Oncology and Radiotherapy, The First Affiliated Hospital of Bengbu Medical University, Bengbu, China

**Keywords:** sarcopenia, esophageal cancer, chemoradiotherapy, prognosis, adverse events

## Abstract

**Background:**

Sarcopenia is a common indicator of systemic nutritional status in patients with cancer progression. This study investigated the impacts of sarcopenia on adverse effects and prognosis of sarcopenia on patients with esophageal cancer receiving chemoradiotherapy.

**Methods:**

The clinical data of 158 patients with initially diagnosed esophageal cancer who received chemoradiotherapy were collected, and nutritional indexes and inflammatory markers were calculated. The cross-sectional areas of the skeletal muscle, subcutaneous fat and visceral fat were calculated using computed tomography (CT) images of the midpoint of the third lumbar (L3) vertebra. The incidence of adverse events, response evaluation, 1-year and 3-year overall survival (OS) and progression-free survival (PFS) were compared between sarcopenia group and non-sarcopenia groups.

**Results:**

This study included 158 patients, 103 (71.5%) in the sarcopenia group and 45 (28.5%) in the non-sarcopenia group. The last follow-up date was January 31, 2024. The median follow-up time was 36 months for all patients. The chi-square test revealed no significant difference in the incidence of serious adverse events between the two groups. The complete response rates (CR) of patients in the sarcopenia and non-sarcopenia groups 1 month after chemoradiotherapy were 2.7 and 13.3%, respectively, *p* = 0.017, and the difference was statistically significant. Moreover, the objective response rates (ORR) were 38.9 and 60.0%, respectively (χ^2^ = 5.770, *p* = 0.016). The median survival time for all patients was 36 months [95% Confidence Interval CI 24–48]. Univariate analysis (Cox proportional risk model) showed that sarcopenia, KPS score, albumin level, T stage, and N stage were correlated with patients’ OS. Multivariate analysis showed that sarcopenia (Hazard Ratio HR 2.84, 95%CI [1.45–5.57], *p* = 0.002), KPS score, albumin level and N stage were independent prognostic factors for OS.

**Conclusion:**

Sarcopenia reduced OS in patients with EC treated with chemoradiotherapy. It can be used as an independent indicator to predict the OS of such patients, which may help in developing optimal treatment strategies.

## Introduction

Esophageal cancer (EC) is characterized by insidious onset, rapid progression, and limited surgical opportunities, resulting in a 5-year survival rate of <20% (1). Concurrent chemoradiotherapy (CRT) is the standard treatment for patients who are unresectable or refuse surgery (2). China has one of the highest global rates of EC, accounting for over half of all cases (3). A retrospective study by Di Fiore et al. showed that baseline nutritional status predicted treatment response and survival after radical CRT in patients with locally advanced EC (4).

Sarcopenia was first proposed by Rosenberg in 1989 (5). Its occurrence is associated with aging (4), systemic inflammation (6), motor neuron changes (7), and decreased anabolism (8). The prevalence of sarcopenia in EC patients ranges from 26 to 57% (9–11), which may be related to advanced age, eating obstruction, tumor burden and CRT-related dysphagia, nausea and vomiting. Currently, the widely used diagnostic criterion is decreased skeletal muscle mass or decreased skeletal muscle strength (12). It has been previously reported that skeletal muscle mass at the third lumbar (L3) vertebral level is proportional to total body muscle mass (11), and the skeletal muscle index (SMI) at the L3 vertebral level based on computed tomography (CT) is currently the most commonly used diagnostic tool for sarcopenia. Prado et al. proposed that sarcopenia is a strong prognostic factor for respiratory and gastrointestinal solid tumors (11). In patients undergoing EC surgery, sarcopenia is associated with increased surgical complications and decreased overall survival (OS) (9), but few studies have been conducted in patients undergoing CRT.

Studies on patients with gastrointestinal cancer have shown that sarcopenia is significantly associated with the infiltration of neutrophils, monocytes, and lymphocytes (13). In addition, systemic inflammatory markers, monocyte-lymphocyte ratio (LMR), platelet-lymphocyte ratio (PLR) and neutrophil-lymphocyte ratio (NLR), also have prognostic significance in EC (14).

Patients with sarcopenia may be more likely to exhibit severe adverse events and shorter OS. This study aimed to investigate the prevalence of pretreatment sarcopenia in EC patients receiving CRT, and to elucidate the associations between sarcopenia, adverse events, and OS. This study provides a basis for prospective studies on the impact of sarcopenia on patients with esophageal cancer undergoing chemoradiotherapy, and may help in developing optimal treatment strategies.

## Materials and methods

### Patients

**Figure 1:**
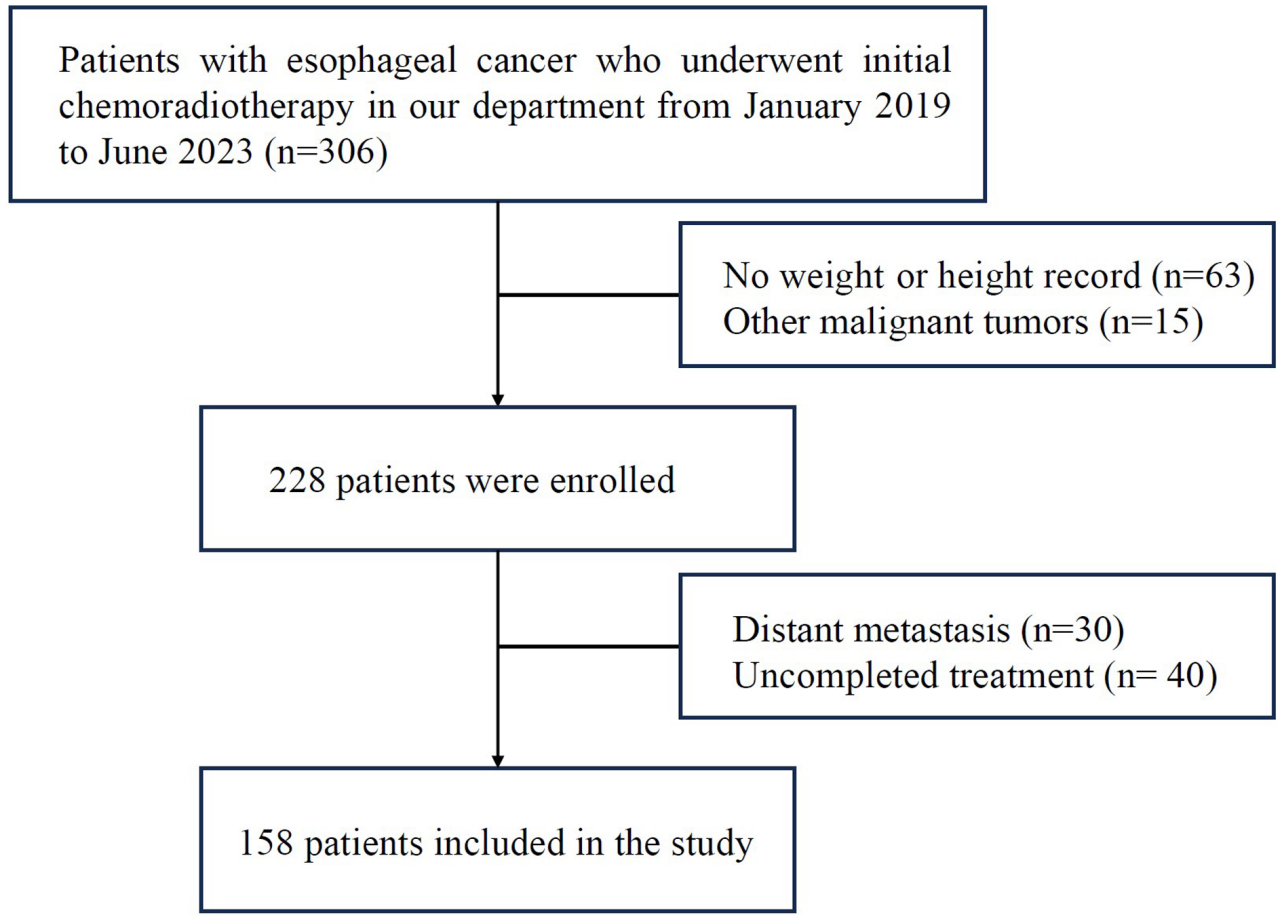
Consort diagram. N number of patients.

A total of 158 CRT patients with EC confirmed by pathology admitted to the First Affiliated Hospital of Bengbu Medical University between January 2019 and June 2023 were enrolled. Inclusion criteria: (1) both sexes, age ≥ 18 years old, life expectancy >3 months; (2) esophageal cancer confirmed by histopathology or cytology; (3) initial concurrent CRT; (4) KPS score ≥ 70. Exclusion criteria: (1) received other antitumor therapy; (2) presence of other malignant tumors; (3) distant metastasis (excluding partial supraclavicular and retroperitoneal lymph node metastasis); (4) presence of complications, like severe cardiopulmonary disease, diabetes or hyperthyroidism and other diseases. The clinical stage was determined by AJCC Version 8 EC staging. A consort flow diagram of patient selection is depicted in Figure 1. This retrospective study has been approved by the Ethics Committee of the First Affiliated Hospital of Bengbu Medical University (ethics approval number: 2023430).

### Data collection

The following baseline clinical data were collected: age, sex, smoking history, alcohol consumption history, KPS score, histological subtype, TNM stage, cancer location, and tumor length. Baseline nutritional parameters were: body weight, body mass index (BMI), history of weight loss before CRT, history of anemia before treatment, serum albumin level, nutritional risk index (NRI), and prognostic nutritional index (PNI). Inflammatory indicators: NLR, PLR, LMR. Treatment characteristics, including chemotherapy regimen and radiotherapy dose, were also collected.

### Treatment

All patients received intensity modulated and conformal radiotherapy (IMRT)/tomo therapy (TOMO) using 6MV-X-rays. The total dose is 50–64 Gy (25–33 Fractions) using the Elekta linear accelerator/Accuray tomo therapy. Chemotherapy regiments mainly include platinum-containing and oral S-1.

### Skeletal muscle, visceral and subcutaneous fat area were measured

**Figure 2:**
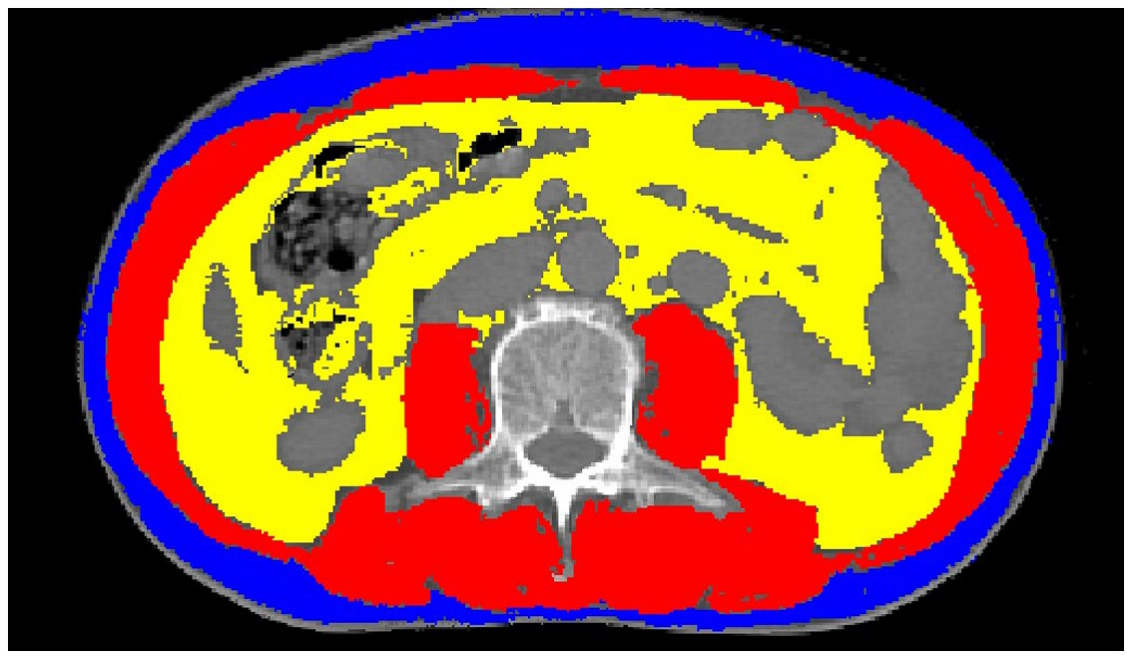
Example of skeletal muscles (red), subcutaneous fat (blue) and visceral fat (yellow), measured on a cross-sectional images of the L3 vertebra.

Combined with simulated positioning CT before radiotherapy, the L3 vertebral midpoint image was derived in medical format. Slice-o-matic 5.0 (Tomovision, Montreal, Canada) was used to analyze the images, and the thresholds for skeletal muscles (including rectus abdominis, transversus abdominis, paddies, paraspinal muscles, internal oblique muscles, and external oblique muscles) were set from −29 to 150 (HUs), visceral fat from −190 to −30 (HUs), and subcutaneous fat from −150 to −50 (HUs), respectively (Figure 2). The cross-sectional area was calculated by semi-automatic delineation (15). The SMI was calculated by dividing the skeletal muscle area by the square height (cm^2^/m^2^). Sarcopenia has been defined as an SMI of ≤41 cm^2^/m^2^ for women and ≤ 53 cm^2^/m^2^ in case of a BMI of ≥ 25 kg/m^2^ or ≤ 43 cm^2^/m^2^ in case of a BMI of < 25 kg/m^2^ for men (16). At the same time, subcutaneous adipose tissue index (SATI, cm^2^/m^2^) and visceral adipose tissue index (VATI, cm^2^/m^2^) were calculated, and skeletal muscle density (SMD, HUs) was measured. Studies on pancreatic cancer suggest that SMD in the L3 vertebra may be more strongly associated with the outcome than SMI (17).

### Clinical definition

According to Chinese criterion (18), anemia is defined as serum hemoglobin level ≤ 12 g/dL in women and ≤ 13 g/dL in men. Serum albumin was divided into normal group and low albumin group according to the critical value of 40.0 g/L determined by our laboratory. The patients were divided into two groups according to the median tumor length of 5 cm and the median age of 74 years. The PNI is calculated as follows: Serum albumin (g/L) + (5 × total lymphocyte count (10^9/L)) (19). NRI: (1.519 × albumin (g/dL)) + 41.7 × (current weight/ideal weight) (20).

### Adverse events and response evaluation

Adverse events during treatment were noted and evaluated using the Common Terminology Standard for Adverse Events (CTCAE) version 5.0. The response evaluation was performed 1 month after the end of CRT according to Response Evaluation Criteria in Solid Tumors (RECIST) version 1.1.

### Follow-up

Patients were first reviewed 1 month after CRT, followed up every 3 months for 2 years, and then every 6 months, with a median follow-up of 28 m as of January 31, 2024. The primary endpoint was OS, defined as the time from CRT initiation to patient death or last follow-up, and the secondary endpoint was progression-free survival (PFS), defined as the time from CRT initiation to disease recurrence or metastasis or last follow-up date.

### Statistical analysis

SPSS 25.0 (IBM Corporation, Armonk, NY, USA) was used for statistical analysis, Fisher precision test and Pearson Chi-square test were used to compare the classified data, and independent sample *t* test or Mann–Whitney *U* test was used for quantitative data. OS and PFS were calculated by Kaplan–Meier curve, and differences between groups were tested by log-rank. Univariate and multivariate analyses were performed using Cox regression model. After univariate analysis, variables with *p* < 0.1 were included in multivariate analysis, *p* < 0.05 was considered statistically significant, and all tests were bilateral.

## Results

### Patient characteristics

A total of 158 patients were included, and the baseline characteristics were shown in Table 1. Comparison between sarcopenic and non-sarcopenic patients. Among them, 45 cases (28.5%) were sarcopenia, 113 cases (71.5%) were non-sarcopenia, male (74.1%), squamous cell carcinoma (94.3%) accounted for the majority. Age ranged from 52 to 86 years (median: 74). The median tumor length was 5 cm. There was no significant difference in baseline characteristics except for BMI and SMD, and BMI in the sarcopenia group was lower than that in the non-sarcopenia group (HR 20.8, 95%CI [19.1–23.1] vs. HR 22.2, 95%CI [20.9–23.5] kg/m^2^, *p* = 0.002). SMD was also lower (31.85 ± 7.41 vs. 35.95 ± 6.77 HUs, *p* = 0.002). There were no statistically significant differences in the inflammatory markers NLR, PLR, or LMR between the two groups, as shown in Table 2.

**Table 1:** Comparison between sarcopenic and non-sarcopenic patients.

Characteristics	Non-sarcopenia	Sarcopenia	*p*
	*n* = 45 (28.5%)	*n* = 113 (71.5%)	
Age, years			0.158
≤74	28 (62.2%)	55 (48.7%)	
>74	17 (37.8%)	58 (51.3%)	
Gender			0.785
Female	11 (24.4%)	30 (26.5%)	
Male	34 (75.6%)	83 (73.5%)	
KPS score			0.698
<80	4 (8.9%)	8 (7.1%)	
≥80	41 (91.1%)	105 (92.9%)	
Smoking			0.332
Yes	15 (33.3%)	29 (25.7%)	
No	30 (66.7%)	84 (74.3%)	
Alcohol			0.823
Yes	10 (22.2%)	27 (23.9%)	
No	35 (77.8%)	86 (76.1%)	
BMI, kg/m^2^			**0.017**
<18.5	1 (2.2%)	18 (15.9%)	
≥18.5	44 (97.8%)	95 (84.1%)	
Albumin			0.597
<40.0	24 (53.3%)	55 (48.7%)	
≥40.0	21 (46.7%)	58 (51.3%)	
Anemia			0.943
Yes	17 (37.8%)	42 (37.2%)	
No	28 (62.2%)	71 (62.8%)	
NRI			0.262
<100	14 (31.1%)	46 (40.7%)	
≥100	31 (68.9%)	67 (59.3%)	
PNI	48.29 ± 0.84	48.66 ± 0.49	0.692
Histological subtype			0.715
SCC	42 (93.3%)	107 (94.7%)	
Other	3 (6.7%)	6 (5.3%)	
Tumor location			0.691
Neck/upper	15 (33.3%)	34 (30.1%)	
Middle/lower	30 (66.7%)	79 (69.9%)	
Length (cm)			0.463
≤5	26 (57.8%)	58 (51.3%)	
>5	19 (42.2%)	55 (48.7%)	
T			0.279
1–2	10 (22.2%)	17 (15.0%)	
3–4	35 (77.8%)	96 (85.0%)	
N			0.296
0–1	28 (62.2%)	80 (70.8%)	
2–3	17 (37.8%)	33 (29.2%)	
M			0.064
0	44 (97.8%)	100 (88.5%)	
1	1 (2.2%)	13 (11.5%)	
TNM			0.411
I-II	17 (37.8%)	35 (31.0%)	
III-IV	28 (62.2%)	78 (69.0%)	
Weight loss (%)			0.064
<5	43 (95.6%)	96 (85.0%)	
≥5	2 (4.4%)	17 (15.0%)	
Nutrition support			0.196
Yes	23 (51.1%)	45 (39.8%)	
No	22 (48.9%)	68 (60.2%)	
Dose (Gy)			1.000
<54	2 (4.4%)	7 (6.2%)	
≥54	43 (95.6%)	106 (93.8%)	
SMI	47.14 ± 4.27	36.09 ± 5.02	**< 0.001**
SMD (HUs)	35.95 ± 6.77	31.85 ± 7.41	**0.002**
SATI	27.57 [19.51, 45.02]	24.84 [16.68, 35.86]	0.737
VATI	25.81 [10.38, 43.99]	16.67 [7.85, 33.89]	0.755

Bold values indicates the *p* value of less than 0.05.

**Table 2:** Comparison of inflammatory indicators.

Inflammatory index	Non-sarcopenia	Sarcopenia	*Z*	*p*
	*n* = 45	*n* = 113		
Neutrophil	3.36 [2.75, 4.25]	3.79 [2.77, 4.64]	−0.730	0.465
Lymphocyte	1.68 [1.35, 1.99]	1.55 [1.25, 1.97]	−0.699	0.484
Platelet	216.18 [181.50, 256.00]	207 [167.50, 263.50]	−0.318	0.751
NLR	2.22 [1.57, 2.98]	2.23 [1.79, 3.21]	−0.724	0.469
PLR	124.46 [104.11, 161.53]	139.41 [97.72, 178.85]	−0.410	0.682
LMR	4.09 [2.83, 4.86]	3.78 [2.84, 4.80]	−0.655	0.513

### Adverse events and response evaluation

Treatment-related adverse events during CRT were shown in Table 3. There was no statistically significant difference in the incidence of serious adverse events between the two groups. However, radiation esophagitis (85.8% vs. 64.4%, *p* = 0.003) and thrombocytopenia (43.4% vs. 24.4%, *p* = 0.030) were more common in sarcopenia patients than in non-sarcopenia patients. The objective response rate (ORR) in the sarcopenia group was significantly lower than that in the non-sarcopenia group (38.9% vs. 60.0%, *p* = 0.016). In addition, complete response rate (CR) tended to be lower in the sarcopenia group (2.7% vs. 13.3%, *p* = 0.017), as shown in Table 4.

**Table 3:** Comparison of adverse events.

Adverse events	Non-sarcopenia		Sarcopenia		*χ* ^2^	*p*
	> = 3 grade	<3grade	> = 3 grade	<3grade		
Leukopenia	9 (20.0%)	36 (80.0%)	16 (14.2%)	97 (85.8%)	0.824	0.364
Neutrophilic granulocytopenia	7 (15.6%)	38 (84.4%)	10 (8.8%)	103 (91.2%)	0.890	0.346
Thrombocytopenia	0	45 (100.0%)	4 (3.5%)	109 (96.5%)	0.515	0.473
Hemoglobinemia	0	0	0	0		
Nausea /vomiting	0	0	0	0		
Radiation esophagitis	0	0	0	0		
Esophageal stenosis	0	45 (100.0%)	1 (0.9%)	112 (99.1%)	0.000	1.000
Radiation pneumonia	0	45 (100.0%)	5 (4.4%)	108 (95.6%)	0.866	0.352
Pulmonary infection	0	0	0	0		

**Table 4:** Comparison of response evaluation.

	CR	PR	SD	PD	ORR
Non-sarcopenia (*n* = 45)	6 (13.3%)	21 (46.7%)	18 (40.0%)	0 (0.0%)	27 (60.0%)
Sarcopenia (*n* = 113)	3 (2.7%)	41 (36.3%)	67 (59.3%)	2 (1.8%)	44 (38.9%)
*χ* ^2^					5.770
*p*	**0.017**				**0.016**

Bold values indicates the *p* value of less than 0.05.

### Survival analysis

**Figure 3:**
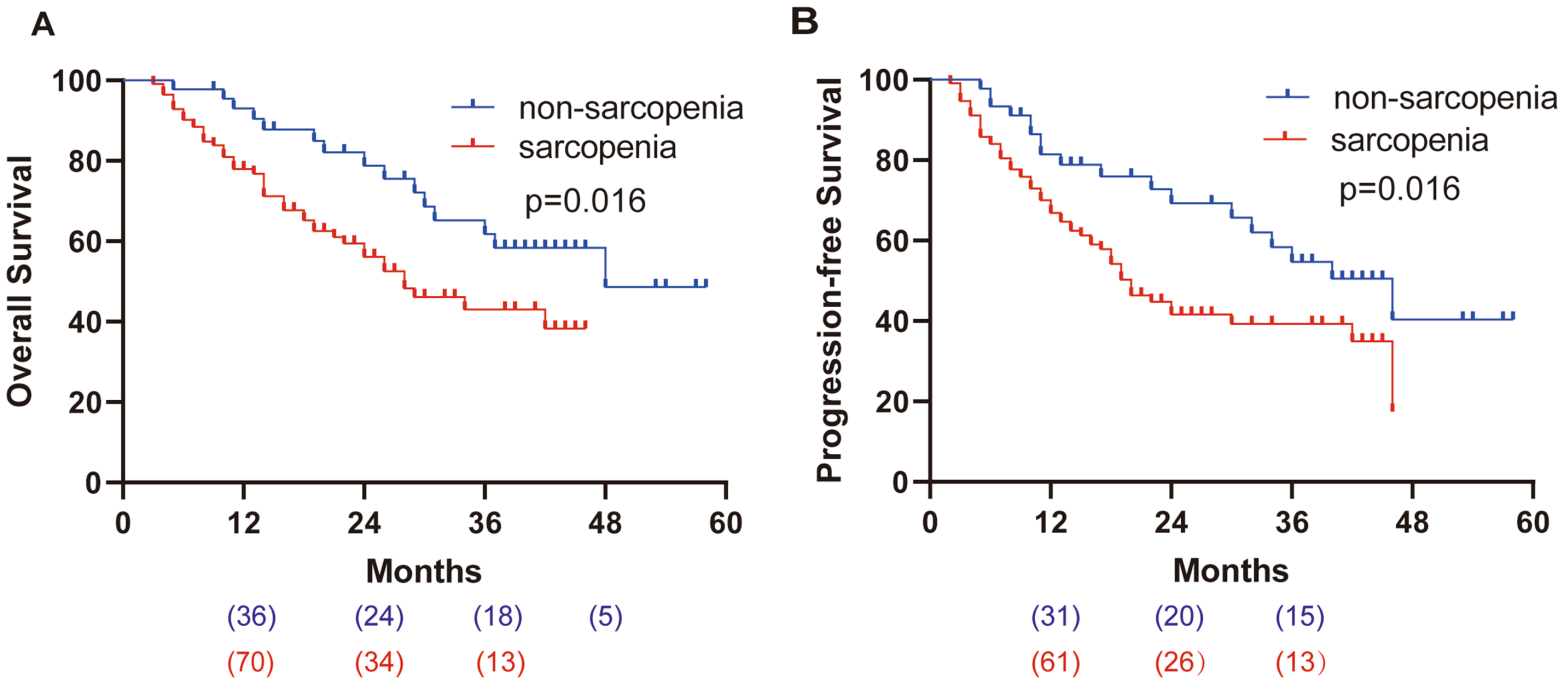
Kaplan–Meier survival curves. **(A)** OS of patients, HR = 2.047. **(B)** PFS of patients, HR = 1.896.

The total median OS was 36 m, as shown in Figure 3A. The median OS in the sarcopenia group and the non-sarcopenia group were 28 m and 48 m, respectively; the 1-year OS rates were 77.9% and 93.0%, and the 3-year OS rate was 43.1% and 61.8%, respectively. The median total PFS was 24 m (Figure 3B). The median PFS in the sarcopenia group and the non-sarcopenia group were 20 m and 46 m, respectively. The 1-year PFS rates were 66.9% and 81.5%, and the 3-year PFS rates were 39.3% and 54.7%, respectively.

### Univariate and multivariate analyses of OS

Univariate analysis showed that (Table 5), KPS score < 80 (HR 2.30, 95%CI [1.09–4.84], *p* = 0.028), albumin <40.0 g/L (HR 1.68, 95%CI [1.02–2.79], *p* = 0.044), T stage (3-4) (HR 2.73, 95%CI [1.09–6.82], *p* = 0.032), N stage (2-3) (HR 1.88, 95%CI [1.14–3.11], *p* = 0.014), TNM stage (III-IV) (HR 1.68, 95%CI [0.95–2.97], *p* = 0.075), tumor location (middle and lower chest) (HR 1.73, 95%CI [0.97–3.10], *p* = 0.063) and sarcopenia (HR 2.05, 95%CI [1.12–3.74], *p* = 0.020) were the adverse factors of OS. Multivariate analysis showed that (Table 6), KPS score < 80 (HR 2.38, 95%CI [1.10–5.18], *p* = 0.009), albumin <40.0 g/L (HR 1.87, 95%CI [1.10–3.16], *p* = 0.020), N stage (2-3) (HR 2.26, 95%CI [1.19–4.26], *p* = 0.012) and sarcopenia (HR 2.84, 95%CI [1.45–5.57], *p* = 0.002) increased the risk of death.

**Table 5:** Univariate analysis of OS and PFS was performed.

Characteristics	OS			PFS		
	HR	95% CI	*p*	HR	95% CI	*p*
Age (>74/≤74)	1.34	0.82–2.21	0.246	1.15	0.73–1.79	0.554
Sex (Male/Female)	0.97	0.58–1.71	0.906	0.86	0.53–1.41	0.553
KPS (<80/≥80)	2.30	1.09–4.84	**0.028**	2.49	1.28–4.85	**0.007**
Smoking (Yes/No)	1.16	0.67–2.00	0.606	1.15	0.70–1.88	0.589
Alcohol (Yes/No)	0.80	0.43–1.50	0.483	0.84	0.48–1.48	0.541
BMI (<18.5/≥18.5)	1.16	0.55–2.44	0.693	1.08	0.52–2.25	0.841
Lost weight in the last 3 months (≥5%/<5%)	0.91	0.39–2.11	0.822	0.86	0.44–1.67	0.648
Anemia (Yes/No)	1.00	0.60–1.67	0.994	0.95	0.59–1.51	0.812
Albumin (<40.0/≥40.0)	1.68	1.02–2.79	**0.044**	1.84	1.16–2.91	**0.010**
NRI (<100/≥100)	1.04	0.63–1.72	0.879	1.26	0.80–1.98	0.312
LMR (≥3.81/<3.81)	1.04	0.63–1.70	0.880	0.91	0.58–1.42	0.661
Tumor length (>5/≤5)	1.33	0.81–2.19	0.259	1.25	0.80–1.96	0.329
T stage (3–4/1–2)	2.73	1.09–6.82	**0.032**	2.58	1.18–5.63	**0.017**
N stage (2–3/0–1)	1.88	1.14–3.11	**0.014**	1.68	1.07–2.65	**0.026**
M stage (1/0)	1.52	0.66–3.54	0.328	1.54	0.70–3.35	0.281
TNM stage (III-IV/I-II)	1.68	0.95–2.97	0.075	1.67	1.00–2.78	0.050
Tumor location (middle and lower chest/neck and upper chest)	1.73	0.97–3.10	0.063	1.46	0.88–2.40	0.144
Tissue type (other/SCC)	1.04	0.37–2.89	0.946	1.47	0.64–3.39	0.366
Radiotherapy dose (≥54/<54)	0.55	0.20–1.53	0.253	0.60	0.24–1.49	0.273
Nutritional support (No/Yes)	0.92	0.56–1.52	0.745	1.08	0.69–1.71	0.731
Sarcopenia (Yes/No)	2.05	1.12–3.74	**0.020**	1.90	1.11–3.25	**0.020**
SMD (≤33/>33)	1.20	0.73–1.99	0.472	1.11	0.70–1.75	0.658
SATI (≤25.2/>25.2)	0.87	0.53–1.42	0.570	0.92	0.59–1.45	0.728
VATI (≤20.6/>20.6)	0.84	0.49–1.43	0.516	0.86	0.53–1.39	0.537

Bold values indicates the *p* value of less than 0.05.

**Table 6:** Multivariate analysis of OS and PFS.

Characteristics	OS			PFS		
	HR	95% CI	*p*	HR	95% CI	*p*
KPS (<80/≥80)	2.38	1.10–5.18	**0.028**	2.55	1.26–5.13	**0.009**
Albumin (<40.0/≥40.0)	1.87	1.10–3.16	**0.020**	1.96	1.22–3.17	**0.006**
N stage (2–3/0–1)	2.26	1.19–4.26	**0.012**	1.86	1.05–3.29	**0.033**
Sarcopenia (Yes/No)	2.84	1.45–5.57	**0.002**	2.53	1.38–4.61	**0.003**

Bold values indicates the *p* value of less than 0.05.

### Univariate and multivariate analyses of FPS

Univariate analysis showed that (Table 5), KPS < 80 (HR 2.49, 95%CI [1.28–4.85], *p* = 0.007), albumin <40.0 g/L (HR 1.84, 95%CI [1.16–2.91], *p* = 0.010), T stage (3-4) (HR 2.58, 95%CI [1.18–5.63], *p* = 0.017), N stage (2-3) (HR 1.68, 95%CI [1.07–2.65], *p* = 0.026), TNM stage (III-IV) (HR 1.67, 95%CI [1.00–2.78], *p* = 0.050) and sarcopenia (HR 1.90, 95%CI [1.11–3.25], *p* = 0.020) were adverse factors for PFS. Multivariate analysis showed that (Table 6), KPS score < 80 (HR 2.56, 95%CI [1.27–5.16], *p* = 0.009), albumin <40.0 g/L (HR 1.98, 95%CI [1.23–3.18], *p* = 0.005), N stage (2-3) (HR 1.94, 95%CI [1.17–3.20], *p* = 0.010) and sarcopenia (HR 2.54, 95%CI [1.40–4.63], *p* = 0.002) lived for a shorter PFS time.

## Discussion

Our research showed that among the 158 EC patients who received CRT, the incidence of radiation esophagitis and thrombocytopenia was higher in patients with sarcopenia, but the incidence of serious adverse events was not increased. Meanwhile, the ORR and CR rates in the sarcopenia group tend to be lower. In addition, sarcopenia, serum albumin <40 g/L, N 2–3 stage, and lower KPS score were independently associated with a poor OS.

According to the diagnostic cut-off value of Martin L et al. (16), the prevalence of sarcopenia in EC in this study was 71.5%, which was higher than previous data (26%–57%), but was generally consistent with local studies (77%–80%) (21, 22), which may be due to ethnic differences. Studies have shown that the muscle mass of the Chinese population is about 17% lower than that of the European population (23). Moreover, it has been suggested that African-Americans present more muscle mass than Caucasian, Asian or Hispanic subjects (24, 25).

In this study, some patients were treated with TOMO due to the too long or wide range of lesions and high treatment requirements, which is different from previous studies. One study has demonstrated that TOMO seems to be significantly better than IMRT in protecting normal tissues; however, there is no significant difference in survival outcomes between the two groups (26).

Sarcopenia has been reported to be associated with a high risk of adverse events during chemotherapy for metastatic breast and colorectal cancer (27, 28). Panje et al. (29) found in their study of locally advanced EC receiving neoadjuvant CRT that the incidence of grade ≥ 3 adverse events in patients with sarcopenia was significantly higher than that in patients without sarcopenia (83.3% vs. 52.4%). Olmez et al. showed that there was no significant difference between the two groups in terms of adverse events in rectal cancer patients receiving synchronous CRT (30). A single-center study of locally advanced EC receiving CRT by Sato et al. (31) showed no statistically significant incidence of serious adverse events in the two groups. There are few studies on the adverse events of sarcopenia on CRT in EC patients, and more studies are still needed. The lack of a significant difference between the two groups in our study could be due to the high prevalence of sarcopenia and the low prevalence of serious adverse effects.

Inflammation plays an important role in cancer progression and prognosis (32), and one study has shown that higher LMR leads to a better clinical response and prognosis in patients with locally advanced EC receiving definitive CRT (33). However, in this study, LMR was not associated with prognosis. It has been noted that the strength of the relationship between higher levels of circulating inflammatory markers and lower skeletal muscle strength and muscle mass varies by population and gender (34). The study was a retrospective study, which may be subject to selection bias and information bias. In addition, the inflammatory markers in this study may not fully reflect the inflammatory state, and future studies should comprehensively analyze with other inflammatory markers, such as IL-6 and TNF-*α*.

The incidence of malnutrition in EC patients before treatment due to mechanical obstruction is as high as 79% (35). In this study, there were no significant differences in nutritional status except for BMI and SMD (albumin, PNI, NRI, SATI, and VATI) between the two groups. Therefore, sarcopenia, as a common indicator reflecting the systemic nutritional status of patients with cancer progression, could be an independent indicator affecting the long-term prognosis of EC patients after CRT. Sato et al. (31) found in EC patients who received radical CRT that the 3-year survival rate of patients with sarcopenia was only 36.95%, which was significantly lower than 63.9% of the non-sarcopenia group. In our study, the 3-year survival rate was 43.1% in the sarcopenia group and 61.8% in the non-sarcopenia group, which was similar to the results. In univariate and multivariate analyses, albumin was a predictor of OS, which was consistent with the results of previous studies (36). Moreover, N stage was also an independent prognostic factor for OS, which fits well with previous studies of esophageal squamous cell carcinoma patients treated with definitive CRT (37).

The study was retrospective and therefore has some limitations. Some patients with tumor progression received molecular targeted therapy or PD-1 immune checkpoint inhibitor treatment during the follow-up period, which may affect the final survival outcome. Therefore, prospective studies with large samples are still needed to confirm it in the future. A systematic review assessing the relationship between protein intake and maintenance of muscle mass in patients undergoing radiotherapy for head and neck cancer, lung cancer, and EC showed that the intake needed to prevent loss of muscle mass during radiotherapy was higher than commonly recommended (>1.4 g/kg vs. 1.2 g/kg) (38). Ma et al. (39) showed that SMI was significantly reduced in EC patients after CRT, indicating significant skeletal muscle loss during CRT. However, due to the lack of post-treatment imaging data, we were unable to analyze changes in skeletal muscle mass in patients treated with CRT, and the usefulness of nutritional support in patients with sarcopenia remains unclear.

In conclusion, lower KPS, BMI < 18.5 kg/m^2^, and lower SMD are common risk factors for sarcopenia in EC patients. Sarcopenia, which reduces the response of treatment and OS rate, is a poor prognostic indicator of CRT in EC patients and may help to provide optimal intervention strategies during treatment.

## Data Availability

The raw data supporting the conclusions of this article will be made available by the authors, without undue reservation.
